# 4-Hydroxyglutamate is a novel predictor of pre-eclampsia

**DOI:** 10.1093/ije/dyz098

**Published:** 2019-05-16

**Authors:** Ulla Sovio, Nancy McBride, Angela M Wood, Katya L Masconi, Emma Cook, Francesca Gaccioli, D Stephen Charnock-Jones, Debbie A Lawlor, Gordon C S Smith

**Affiliations:** 1 Department of Obstetrics and Gynaecology, University of Cambridge; NIHR Cambridge Biomedical Research Centre, Cambridge, UK; 2 Centre for Trophoblast Research (CTR), Department of Physiology, Development and Neuroscience, University of Cambridge, Cambridge, UK; 3 NIHR Bristol Biomedical Research Centre, Bristol, UK; 4 MRC Integrative Epidemiology Unit, at the University of Bristol, Bristol, UK; 5 Population Health Sciences, Bristol Medical School, Bristol, UK; 6 Department of Public Health and Primary Care, University of Cambridge, Cambridge, UK

**Keywords:** Metabolomics, risk prediction, pre-eclampsia, pregnancy, cohort study

## Abstract

**Background:**

Pre-term pre-eclampsia is a major cause of maternal and perinatal morbidity and mortality worldwide. A multi-centre randomized–controlled trial has shown that first-trimester screening followed by treatment of high-risk women with aspirin reduces the risk of pre-term pre-eclampsia. However, the biomarkers currently employed in risk prediction are only weakly associated with the outcome.

**Methods:**

We conducted a case–cohort study within the Pregnancy Outcome Prediction study to analyse untargeted maternal serum metabolomics in samples from 12, 20, 28 and 36 weeks of gestational age (wkGA) in women with pre-eclampsia delivering at term (*n* = 165) and pre-term (*n* = 29), plus a random sample of the cohort (*n* = 325). We used longitudinal linear mixed models to identify candidate metabolites at 20/28 wkGA that differed by term pre-eclampsia status. Candidates were validated using measurements at 36 wkGA in the same women. We then tested the association between the 12-, 20- and 28-wkGA measurements and pre-term pre-eclampsia. We externally validated the association using 24- to 28-wkGA samples from the Born in Bradford study (25 cases and 953 controls).

**Results:**

We identified 100 metabolites that differed most at 20/28 wkGA in term pre-eclampsia. Thirty-three of these were validated (*P *<* *0.0005) at 36 wkGA. 4-Hydroxyglutamate and C-glycosyltryptophan were independently predictive at 36 wkGA of term pre-eclampsia. 4-Hydroxyglutamate was also predictive (area under the receiver operating characteristic curve, 95% confidence interval) of pre-term pre-eclampsia at 12 (0.673, 0.558–0.787), 20 (0.731, 0.657–0.806) and 28 wkGA (0.733, 0.627–0.839). The predictive ability of 4-hydroxyglutamate at 12 wkGA was stronger than two existing protein biomarkers, namely PAPP-A (0.567, 0.439–0.695) and placenta growth factor (0.589, 0.463–0.714). Finally, 4-hydroxyglutamate at 24–28 wkGA was positively associated with pre-eclampsia (term or pre-term) among women from the Born in Bradford study.

**Conclusions:**

4-hydroxyglutamate is a novel biochemical predictor of pre-eclampsia that provides better first-trimester prediction of pre-term disease than currently employed protein biomarkers.

**Key Messages**

- Discovery of new biomarkers associated with pre-term pre-eclampsia could lead to improved risk prediction and identification of women who are most likely to benefit from preventive treatment.
- We conducted a case–cohort study to identify candidate metabolites from maternal serum that are predictive of pre-eclampsia and we validated the findings internally and in an external cohort.
- We identified a single metabolite, 4-hydroxyglutamate, which is strongly associated with pre-eclampsia and the association is similar in two demographically different cohorts.
- 4-Hydroxyglutamate provides better first-trimester prediction of pre-term pre-eclampsia than currently employed biomarkers.

## Introduction

Pre-eclampsia is a multisystem disorder of human pregnancy manifested by acquired hypertension plus involvement of other systems including the kidney, liver, brain and platelets.^1^ Pre-eclampsia is a major determinant of the global burden of disease through effects on the mother and infant. Death of the mother is primarily a feature of low- and middle-income countries^2^ but hypertensive disorders were a common cause of obstetric Intensive Therapy Unit admissions in a UK-based study.^3^ Hypertensive disorders of pregnancy are a major cause of perinatal morbidity and mortality worldwide, being a factor in about 10–20% of perinatal deaths^4^ and one of the major causes of iatrogenic prematurity.^5^

The ASPRE trial demonstrated that use of 150 mg of aspirin, given daily and commenced at between 11 and 14 weeks of gestational age (wkGA), reduced the risk of pre-term delivery due to pre-eclampsia by 62% in women who screened as high-risk in the first trimester.^6^ The ASPRE trial used a predictive algorithm that included maternal serum levels of placenta growth factor (PlGF) and pregnancy-associated plasma protein A (PAPP-A) in addition to maternal factors, mean arterial pressure and uterine artery pulsatility index. However, the rate of pre-term birth due to pre-eclampsia was only 4.3% in the placebo group, reflecting a high false-positive rate. The aim of the present study was to identify novel biochemical predictors of pre-eclampsia. We performed a case–cohort study using samples from the Pregnancy Outcome Prediction (POP) study and performed metabolomic profiling of maternal serum samples collected at 12, 20, 28 and 36 wkGA, comparing women who subsequently delivered with a diagnosis of pre-eclampsia with a random sample of women who did not have pre-eclampsia. We used a case–control study of samples from Born in Bradford (BiB), a highly demographically different cohort, for external validation.

## Methods

### Identification and validation of metabolic markers

**Figure 1.**
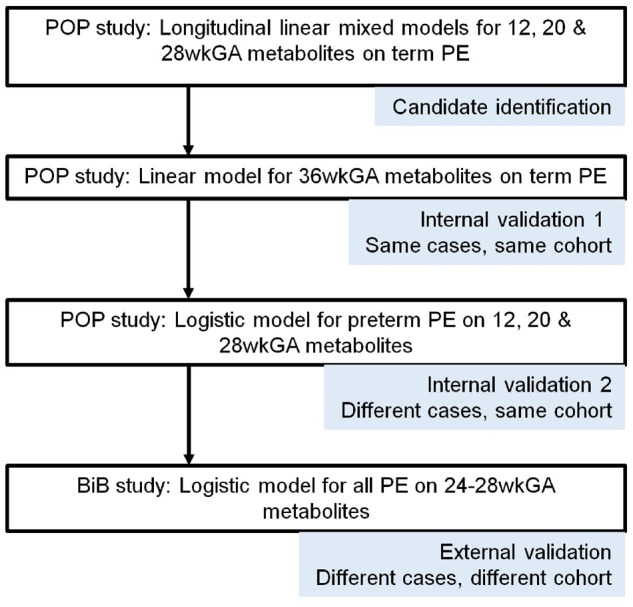
Schematic outline for identification and validation of predictive metabolites. PE, pre-eclampsia; wkGA, weeks of gestational age; POP, Pregnancy Outcome Prediction; BiB, Born in Bradford.

Figure 1 illustrates an overview of the approach employed to identify and validate novel metabolic predictors of pre-eclampsia. Identification of candidate metabolites was followed by two steps of internal validation, the first using repeated metabolite measurements from different gestational ages from the same cases and the second using different cases. This was followed by validation in an external cohort.

### Study design

The POP study was a prospective cohort study of unselected nulliparous women with a singleton pregnancy attending the Rosie Hospital, Cambridge, UK, between January 2008 and July 2012. The design has been previously described.^7–9^ Participants had phlebotomy and fetal biometry at 12, 20, 28 and 36 wkGA and outcome data were retrieved through individual review of each patient’s case record and by linkage to electronic databases. Ethical approval was obtained from the Cambridgeshire 2 Research Ethics Committee (reference number 07/H0308/163). All study participants gave written informed consent. This study is reported according to the STARD 2015 guidelines for reporting diagnostic accuracy studies (http://www.stard-statement.org/) and we have included a completed STARD checklist (available as Supplementary data at *IJE* online).

A case–cohort design within the POP study was used in the present analysis (study outline, available as Supplementary data at *IJE* online). In brief, a random sample of the cohort was selected as a comparison group. Two categories of cases were analysed in the present study: (i) women who experienced pre-eclampsia delivering at term and (ii) women who experienced pre-eclampsia delivering pre-term. Pre-eclampsia was defined and classified using the 2013 ACOG Guideline.^10^^,^^11^ Term pre-eclampsia cases were compared with women from the random sub-cohort who delivered at term without experiencing any pre-eclampsia. Pre-term cases were compared with the all the women in the random sub-cohort who did not experience pre-term pre-eclampsia. Classification of pre-eclampsia was performed blind to the results of the biochemical analyses.

### External validation: the BiB cohort

The BiB cohort is described in detail elsewhere.^12^^,^^13^ Recruitment to this study was conducted between 2007 and 2011 and included 12 453 women having 13 776 pregnancies. The study was initiated in light of concerns about high rates of childhood morbidity and mortality in Bradford (a multi-ethnic city in the north of England with a high level of socio-economic deprivation). The BiB study recruited a population that was profoundly different in its demographic characteristics compared with the POP study, in terms of ethnicity (40 vs 93% White European ethnicity, respectively), parity (40 vs 100% nulliparous, respectively) and socio-economic deprivation (68 vs 0% living in an area of the highest quintile of socio-economic deprivation in England, respectively). All women in the cohort, except those with pre-existing diabetes, were invited to attend for a glucose-tolerance test at 24–28 wkGA, with 85% completing and having valid test data. Pre-eclampsia was defined using all measurements of blood pressure and proteinuria extracted from antenatal records and applying the International Society for the Study of Hypertension in Pregnancy criteria to these.^14^ Those without a documented pre-eclampsia status were excluded from the analysis (*n* = 22).

### Biochemical analyses

Metabolomic analysis was performed by Metabolon (Research Triangle Park, NC, USA), blinded to the patients’ clinical information and pregnancy outcome. In the POP study, analysis batches contained 36 maternal serum samples each and all samples from the same woman were included in the same batch. Batches were designed so that the proportion of samples from cases and controls was the same in all batches. Ultrahigh Performance Liquid Chromatography-Tandem Mass Spectroscopy (UPLC-MS/MS) was used as the analysis platform (metabolomics profiling details, available as Supplementary data at *IJE* online). Metabolite concentrations were quantified using area-under-the-curve of primary MS ions and were expressed as the multiple of the median value for all batches processed on the given day; 1193 untargeted metabolites were measured from each sample: 837 of known structural identity and 356 of unknown structural identity. Measurement of protein levels [PAPP-A, PlGF and fms-like tyrosine kinase 1 (sFlt-1)] was performed on maternal serum using the Roche Cobas e411 immunoassay platform, as previously described.^11^ Samples from the BiB study were sent separately to Metabolon and the approach to analysis was identical to that used in the POP study. Metabolomics analysis was performed on ethylenediamine tetraacetic acid (EDTA) plasma samples from 1000 women, selected randomly from women who had completed the glucose-tolerance test, had stored fasting plasma and were of either White British or Pakistani origin, reflecting the two largest homogenous ethnic groups and comprising 84% of the participants.

### Statistical analysis

Analyses in the POP study were predefined in an analysis plan (available as Supplementary data at *IJE* online). Scaled imputed metabolite values (multiples of the median) were log-transformed. Initial identification of metabolites associated with term pre-eclampsia involved fitting longitudinal linear mixed models for each metabolite using measurements from 12, 20 and 28 wkGA, to generate a difference in the metabolite means and associated *P*-value in the maternal serum at 20 and/or 28 wkGA (composite chi-squared test) comparing term pre-eclampsia cases and controls. We included interaction terms between term pre-eclampsia and gestational age to identify these differences. The metabolites were then ranked by the composite *P*-value at 20/28 wkGA. Excess of low *P*-values was tested using a one-sample Kolmogorov–Smirnov test against the theoretical random distribution of *P*-values between 0 and 1. Initial validation used the 36-wkGA sample in the same women and differences between cases and controls were assessed using a linear-regression model between the metabolite and term pre-eclampsia status. Analysis of the 36-wkGA sample employed a Bonferroni correction to the *P*-value with the aim of minimizing false-positive results. Forward-stepwise logistic regression (*P *<* *0.05 for entry and *P *<* *0.1 for removal) was then used to select independent metabolites for the prediction of term pre-eclampsia, with inclusion of known predictors: maternal age, body mass index (BMI) and the log-transformed sFlt-1:PlGF ratio.

The log-transformed values of the selected metabolites were turned into z (standard deviation) scores to enable direct comparison between the metabolites. Associations between selected metabolites and pre-eclampsia outcomes were reported using odds ratios (ORs) per 1 standard deviation higher metabolite value with 95% confidence intervals (CIs).

Prediction of pre-term pre-eclampsia was assessed using the area under the receiver operating characteristic (ROC) curve (AUC). In the presence of multiple predictors, the AUC was calculated from the predicted probability generated by a multivariable logistic-regression model. The AUC was estimated using 1000-fold bootstrapping to avoid optimism through overfitting.^15^ Assessing the added predictive value of a variable was quantified using the change in the AUC and was also assessed by the likelihood ratio test comparing models with and without the given variable. Sensitivity and positive predictive value were reported for a 10% screen positive rate. Positive and negative predictive values were calculated using weighting of the comparison group by the inverse of the sampling fraction.

The association with known maternal predictors of pre-term pre-eclampsia was quantified using a previously described competing risks time-to-event analytic model.^6^ The maternal variables included in this model were age, height, ethnicity, chronic hypertension, systemic lupus erythematosus or anti-phospholipid syndrome and conception by *in vitro* fertilization (all women were nulliparous and therefore additional coefficients for parous women with or without previous pre-eclampsia were not used). In women without chronic hypertension, weight and diabetes status were also used (family history of pre-eclampsia was not recorded in the POP study and the value was imputed to none). The output of this model is the predicted gestational age of pre-eclampsia (PGAPE), where the risk of pre-eclampsia is higher in women with lower values. PGAPE was calculated using the data available at the 12-wkGA visit and its association with pre-term pre-eclampsia was estimated separately at 12, 20 and 28 wkGA for the subset of women who had a serum sample available at the given gestational time point.

External validation was performed using samples from the BiB cohort. The scaled, imputed metabolite concentrations of 4-hydroxyglutamate were log-transformed to improve normality and scaled to z scores. The association between 4-hydroxyglutamate and pre-eclampsia was reported using OR per 1 standard deviation higher metabolite value with 95% CIs.

All statistical analysis employed Stata 15.1 and R 3.4.4.

## Results

The characteristics of the groups from the POP study are summarized in Table 1. Women who delivered with pre-eclampsia at term were induced more often compared with the control group. Women who delivered with pre-eclampsia pre term had a pre-labour caesarean delivery more often compared with the control group and their babies were smaller at birth. Maternal BMI was higher and education level lower in both groups of cases than in the controls.

**Table 1.** Characteristics of the POP study cohort in the metabolomics analysis of pre-eclampsia

Characteristic	PE term (*N* = 165)	PE pre-term (*N* = 29)	Controls without pre-term PE (*N* = 323)
**Maternal characteristics**			
Age, years	30 (26 to 34)	28 (23 to 33)	30 (27 to 33)
Age stopped FTE ≥21 years	75 (45%)	8 (28%)	177 (55%)
Missing	5 (3%)	1 (3%)	2 (1%)
Height, cm	165 (160 to 168)	163 (158 to 166)	165 (161 to 169)
BMI, kg/m^2^	26 (23 to 32)	28 (26 to 30)	24 (22 to 28)
Smoker	6 (4%)	1 (3%)	15 (5%)
Any alcohol consumption	7 (4%)	0 (0%)	12 (4%)
Deprivation, score	8.91 (5.56 to 13.59)	9.66 (5.68 to 11.85)	8.53 (5.95 to 14.18)
Deprivation, rank	25246 (19634 to 29324)	24266 (21549 to 29179)	25727 (19039 to 28872)
Deprivation rank quintile			
1 (most deprived)	0 (0%)	0 (0%)	0 (0%)
2	13 (8%)	1 (3%)	20 (6%)
3	26 (16%)	5 (17%)	64 (20%)
4	45 (27%)	13 (45%)	79 (24%)
5 (least deprived)	73 (44%)	10 (34%)	148 (46%)
Missing	8 (5%)	0 (0%)	12 (4%)
White ethnicity	157 (95%)	26 (90%)	302 (94%)
Missing	1 (1%)	1 (4%)	6 (2%)
Married	105 (64%)	22 (76%)	232 (72%)
Diabetes			
Type 1 or type 2 DM	2 (1%)	3 (10%)	0 (0%)
Gestational DM	11 (7%)	1 (3%)	11 (3%)
Essential HT	35 (21%)	12 (41%)	9 (3%)
Pre-existing renal disease	3 (2%)	1 (3%)	2 (1%)
**Birth outcomes**			
Birthweight, g	3390 (3050 to 3760)	2130 (1600 to 2580)	3445 (3100 to 3775)
Birthweight, centile	45 (24 to 67)	17 (8 to 44)	47 (23 to 68)
Gestational age, weeks	39.9 (38.6 to 40.9)	35.3 (33.3 to 36.3)	40.3 (39.3 to 41.3)
Female fetal sex	72 (44%)	16 (55%)	165 (51%)
Induction of labour	106 (64%)	7 (24%)	114 (35%)
Mode of delivery			
Spontaneous vaginal	36 (22%)	7 (24%)	161 (50%)
Assisted vaginal	51 (31%)	0 (0%)	68 (21%)
Intra-partum caesarean	55 (33%)	3 (10%)	59 (18%)
Pre-labour caesarean	22 (13%)	19 (66%)	33 (10%)
Missing	1 (1%)	0 (0%)	2 (1%)

In total, 4212 women completed the POP study. After the exclusion of miscarriages, fetal deaths prior to 23 wkGA and terminations (total *n* = 29) and women who did not have any blood samples available (*n* = 6), 4177 women remained in the cohort and the random sub-cohort (*n* = 325) was selected from this population. Data are expressed as median (IQR) or *n* (%) as appropriate. For fields where there is no category labelled ‘missing’, data were 100% complete.

Maternal age was defined as age at recruitment. All other maternal characteristics were defined by self-report at the 20-wkGA questionnaire, from examination of the clinical case record or linkage to the hospital’s electronic databases. The weight measurement used in the BMI calculation was made at the 12-wkGA visit. Socio-economic status was quantified using the Index of Multiple Deprivation (IMD) 2007,^16^ which is based on census data from the area of the mother’s postcode. Deprivation score is the combined sum of the weighted, exponentially transformed domain rank of the domain score and higher values indicate more deprivation. Conversely, the most deprived area has the lowest rank (= 1) and the least deprived area has the highest rank (= 32 482). A national reference distribution from 2010^17^ has been used to analyse the rank in quintiles (1 = most deprived, 5 = least deprived), enabling a comparison with the Born in Bradford study. Pre-eclampsia was defined on the basis of the 2013 ACOG criteria. FTE, full-time education; BMI, body mass index; DM, diabetes mellitus; PE, pre-eclampsia; HT, hypertension.

**Figure 2.**
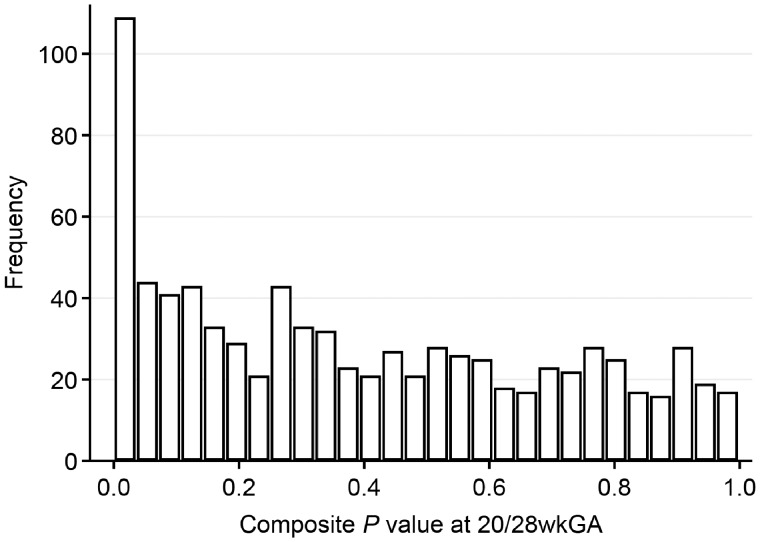
Distribution of *P*-values from the composite chi-squared test (two-sided) for the measurements at 20/28 weeks of gestational age (wkGA). The *P*-values of 829 metabolites with a known structural identity were calculated from the test for interaction between term pre-eclampsia and gestational age.

We excluded 356 metabolites of unknown structural identity and 8 xenobiotic metabolites that had too little variation to be analysed, leaving 829 metabolites for the analysis. A plot of the composite *P*-values indicated an excess of low values (Kolmogorov–Smirnov test *P *<* *0.001), suggesting that there was an association between the metabolite values at 20/28 wkGA and the risk of term pre-eclampsia (Figure 2). The 100 metabolites with the lowest *P*-values (<0.032) were selected for further study.

Using a Bonferroni-corrected threshold (i.e. *P *<* *0.0005), 33 of the 100 metabolites were different between term cases and controls at 36 wkGA (Supplementary Table 1, available as Supplementary data at *IJE* online). Many of these metabolites were correlated (Supplementary Table 2, available as Supplementary data at *IJE* online). The variables independently associated with term pre-eclampsia in the forward-stepwise logistic regression were maternal BMI and age, the sFlt-1:PlGF ratio, 4-hydroxyglutamate, C-glycosyltryptophan and 1-palmitoyl-2-linoleoyl-GPE (16:0/18:2).

We then determined the extent to which each one of them at 36 wkGA added to the prediction achieved by the sFlt1:PlGF ratio, a currently employed biomarker for pre-eclampsia. The AUC for the sFlt-1:PlGF ratio at 36 wkGA on its own was 0.806. The single metabolite that enhanced prediction most was 4-hydroxyglutamate, which increased the AUC by 0.028 (AUC = 0.834). The second most informative metabolite, C-glycosyltryptophan, further improved the AUC by 0.014 (AUC = 0.848). The remaining metabolite, 1-palmitoyl-2-linoleoyl-GPE (16:0/18:2), did not further improve the AUC (increase = 0.001, AUC = 0.849).

**Figure 3.**
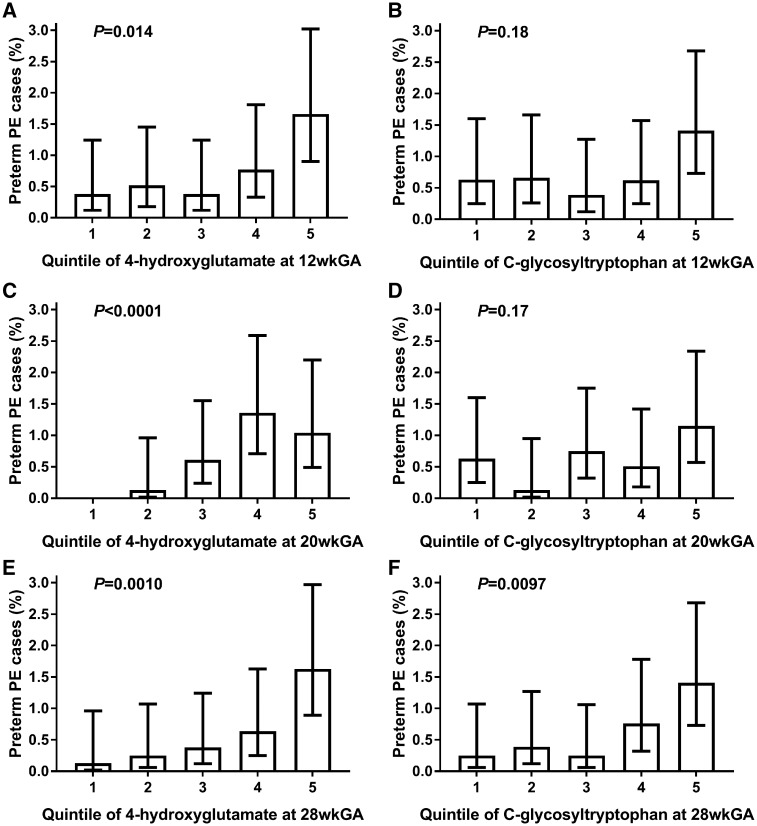
The proportion of pre-term pre-eclampsia cases (95% confidence interval) by the quintile of 4-hydroxyglutamate and C-glycosyltryptophan at 12, 20 and 28 wkGA. (**A**) 4-hydroxyglutamate at 12 wkGA; (**B**) C-glycosyltryptophan at 12 wkGA; (**C**) 4-hydroxyglutamate at 20 wkGA; (**D**) C-glycosyltryptophan at 20 wkGA; (**E**) 4-hydroxyglutamate at 28 wkGA; (**F**) C-glycosyltryptophan at 28 wkGA. The non-cases were weighted by the inverse of the random sub-cohort sampling fraction (= 12.85) to obtain the proportion of cases of pre-term pre-eclampsia. There were no cases in the first quintile of 4-hydroxyglutamate at 20 wkGA and therefore the 95% confidence interval could not be calculated. Two-sided logistic-regression *P*-values are given for the linear trend between the quintile and log-odds of pre-term pre-eclampsia. PE, pre-eclampsia; wkGA, weeks of gestational age.

We sought to evaluate these two candidates further as predictors of pre-eclampsia resulting in pre-term delivery. Plotting each metabolite by quintiles (Figure 3) revealed strong positive associations between 4-hydroxyglutamate measured at 12, 20 and 28 wkGA and pre-term pre-eclampsia, whereas C-glycosyltryptophan was only strongly associated with pre-term pre-eclampsia when measured at 28 wkGA. When the two metabolites were adjusted for each other, the strong positive associations between 4-hydroxyglutamate at 12, 20 and 28 wkGA and the risk of pre-term pre-eclampsia remained (Table 2). In relation to C-glycosyltryptophan and pre-term pre-eclampsia, there was no sufficient evidence for an association at 12 or 20 wkGA, but there was still a strong association at 28 wkGA. Therefore, in further analysis, we focused on the predictive ability of 4-hydroxyglutamate at 12 and 20 wkGA and of both 4-hydroxyglutamate and C-glycosyltryptophan at 28 wkGA.

**Table 2.** Odds ratios (95% confidence intervals) and *P*-value of the unadjusted and adjusted metabolite measurements (a) at 12, 20 and 28 wkGA in relation to pre-term pre-eclampsia (*n* = 29) and (b) at 36 wkGA in relation to term pre-eclampsia (*n* = 165) in the case–cohort sample (total *n* = 504)

		Odds ratios (95% confidence intervals) and *P*-value			
Metabolite	Model	12 wkGA and pre-term PE	20 wkGA and pre-term PE	28 wkGA and pre-term PE	36 wkGA and term PE
4-hydroxyglutamate	Unadjusted	2.04 (1.37 to 3.04)	2.38 (1.46 to 3.88)	2.37 (1.53 to 3.66)	2.24 (1.70 to 2.96)
		*P*=0.0004	*P*=0.0005	*P*=0.0001	*P*<0.0001
	Adjusted	1.99 (1.33 to 2.98)	2.40 (1.45 to 3.98)	2.05 (1.29 to 3.26)	1.87 (1.42 to 2.47)
		*P*=0.0009	*P*=0.0007	*P*=0.0024	*P*<0.0001
C-glycosyltryptophan	Unadjusted	1.48 (0.97 to 2.24)	1.58 (1.08 to 2.33)	2.26 (1.48 to 3.45)	2.67 (2.05 to 3.48)
		*P*=0.067	*P*=0.020	*P*=0.0001	*P*<0.0001
	Adjusted	1.37 (0.88 to 2.13)	1.56 (1.05 to 2.33)	1.98 (1.27 to 3.10)	2.55 (1.92 to 3.38)
		*P*=0.17	*P*=0.029	*P*=0.0027	*P*<0.0001

Odds ratios are given for 1 standard deviation increase in the log-transformed metabolite. Adjusted models are only adjusted for the other metabolite. All *P*-values are two-sided. wkGA, weeks of gestational age; PE, pre-eclampsia.

**Figure 4.**
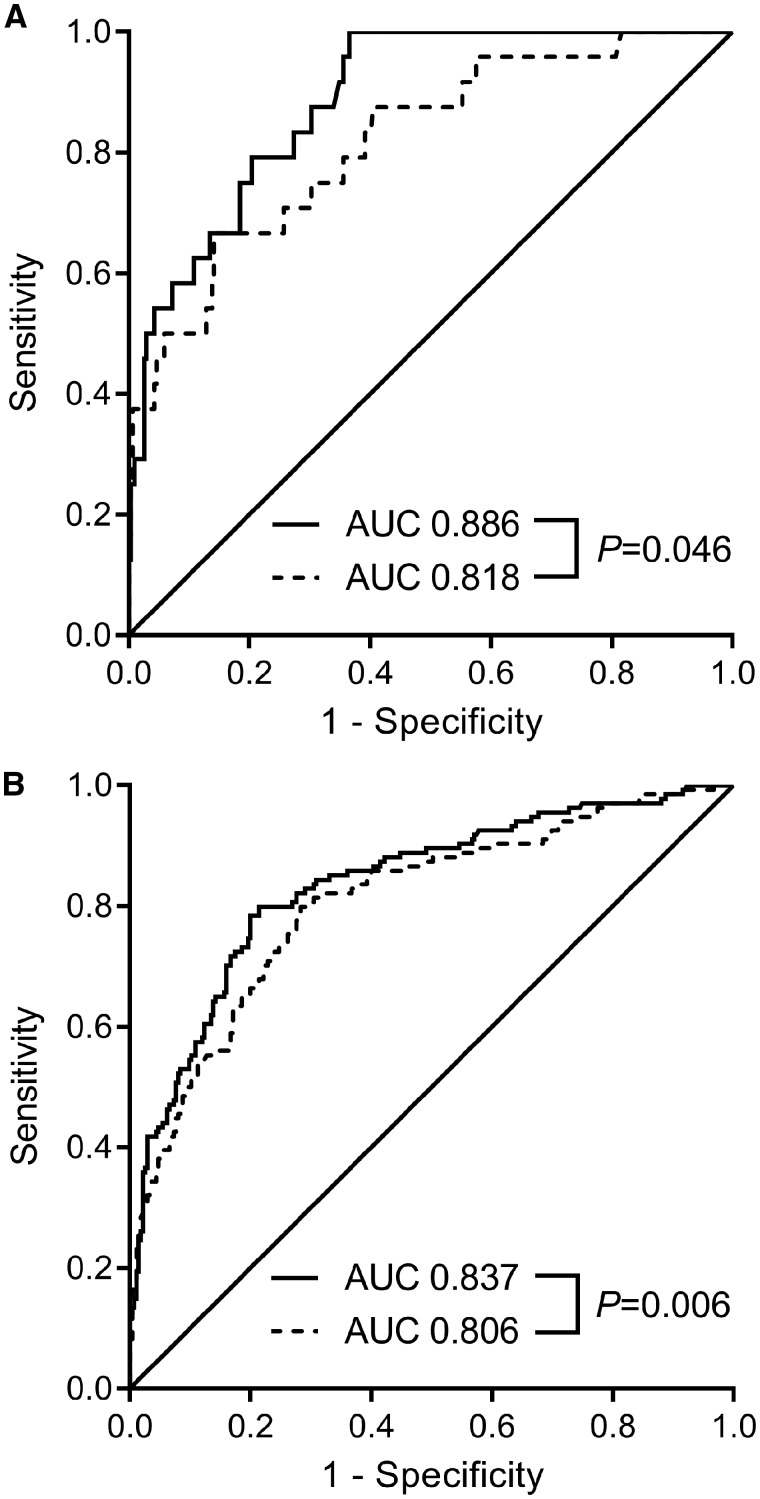
Receiver operating characteristic curve (ROC) analysis of the sFlt-1:PlGF ratio and 4-hydroxyglutamate in relation to pre-term and term pre-eclampsia. The ROC curve for (**A**) addition of 4-hydroxyglutamate to sFlt-1:PlGF ratio at 28 wkGA and pre-term pre-eclampsia and (**B**) addition of 4-hydroxyglutamate to sFlt-1:PlGF ratio at 36 wkGA and term pre-eclampsia. Dashed lines represent the sFlt-1:PlGF ratio measurements on their own and solid lines represent the models that include both sFlt-1:PlGF ratio and 4-hydroxyglutamate. The *P-*value was obtained from the De Long test for equality between the AUCs. AUC, area under the ROC curve; wkGA, weeks of gestational age.

At 12 wkGA, the predictive ability (AUC, 95% CI) of 4-hydroxyglutamate for the risk of pre-term pre-eclampsia (0.673, 0.558–0.787) was stronger than either PAPP-A (0.567, 0.439–0.695) or PlGF (0.589, 0.463–0.714). We then examined the predictive ability of combinations of biomarkers with a previously described model to predict the risk of pre-term pre-eclampsia using maternal characteristics (Table 3),^6^ where the output (PGAPE) was inversely associated with the risk of disease. PGAPE on its own was highly predictive of the risk of pre-term pre-eclampsia (0.863, 0.798–0.927). 4-Hydroxyglutamate enhanced prediction achieved using PGAPE (increase in AUC = 0.019). In contrast, adding PAPP-A and PlGF to models containing only PGAPE or PGAPE and 4-hydroxyglutamate resulted in negligible changes in the AUC. At 20 wkGA, 4-hydroxyglutamate also enhanced prediction of pre-term pre-eclampsia and both 4-hydroxyglutamate and C-glycosyltryptophan enhanced prediction at 28 wkGA (Supplementary Tables 3 and 4, available as Supplementary data at *IJE* online). We have also presented a ROC curve analysis for the addition of 4-hydroxyglutamate to the sFlt-1:PlGF ratio at 28 wkGA in the prediction of pre-term pre-eclampsia and at 36 wkGA in the prediction of term pre-eclampsia (Figure 4).

**Table 3.** Prediction of pre-term pre-eclampsia at 12 wkGA

Model	AUC	Change in AUC	*P*-value^a^
PGAPE + PAPP-A + PlGF + 4-hydroxyglutamate	0.887	–	–
PGAPE + 4-hydroxyglutamate	0.882	–0.005	0.14
PGAPE + PAPP-A + PlGF	0.859	–0.028	0.023
PGAPE	0.863	–0.024	0.035
4-hydroxyglutamate	0.673	–0.214	<0.0001
PAPP-A + PlGF	0.539	–0.348	<0.0001

a Compared with full model using likelihood ratio test of nested logistic-regression models. Where the model included more than one predictor, the AUC was corrected for optimism using 1000-fold bootstrapping.

PGAPE = the predicted gestational age of pre-eclampsia. This is the output of the competing risks time-to-event model employed in the ASPRE study (see Rolnik *et al.*, 2017^6^ for details).

AUC, area under the ROC curve; wkGA, weeks of gestational age; PAPP-A, pregnancy-associated plasma protein A; PlGF, placenta growth factor.

Using a 10% screen positive rate, adding first-trimester 4-hydroxyglutamate levels to PGAPE increased the sensitivity for pre-term pre-eclampsia from 51.7 to 55.2% and increased the positive predictive value from 6.1 to 6.8%, although the CIs were wide (Supplementary Table 5, available as Supplementary data at *IJE* online). Nevertheless, using the point estimates, the effect of adding 4-hydroxyglutamate to PGAPE was to reduce the ratio of false positives to true positives by 11%.

Finally, we performed external validation analysing maternal EDTA plasma metabolomics performed on samples obtained at 24–28 wkGA in the BiB cohort, comparing 25 women who subsequently had a pregnancy complicated by pre-eclampsia and 953 women who did not. The characteristics of the two groups were tabulated (Supplementary Table 6, available as Supplementary data at *IJE* online). We found that 4-hydroxyglutamate levels were higher in women who went on to develop pre-eclampsia (OR = 1.43, one-sided *P *=* *0.029) and the magnitude of the association was comparable to that observed in the POP study between 4-hydroxyglutamate at 28 wkGA and the risk of any subsequent pre-eclampsia (OR = 1.51).

## Discussion

The ASPRE multi-centre randomized–controlled trial reported in 2017 that 150 mg aspirin given at night from 11–14 through to 36 wkGA reduced the risk of pre-term delivery with a diagnosis of pre-eclampsia by 62%. The study’s approach was to target aspirin to women identified as at high risk of pre-term pre-eclampsia as assessed by a model combining maternal characteristics, protein biomarkers and uterine artery Doppler flow velocimetry. The main finding of the present study is that first-trimester maternal serum levels of 4-hydroxyglutamate were more strongly associated with the risk of pre-term pre-eclampsia than the two protein biomarkers employed in the ASPRE algorithm: PAPP-A and PlGF. Moreover, unlike the protein biomarkers, 4-hydroxyglutamate levels were associated with pre-term pre-eclampsia independently of maternal characteristics. To our knowledge, this is the first report of any association between 4-hydroxyglutamate and an acquired human disease.

We used a discovery-based (i.e. untargeted) method to identify the association with 4-hydroxyglutamate. The only previously described use for measurement of 4-hydroxyglutamate in assessing disease risk, to our knowledge, has been to aid the diagnosis of primary hyperoxaluria type 3^18^ and there are very few published data that could shed light on the biological basis for a link with pre-eclampsia. 4-Hydroxyglutamate is formed from 4-hydroxyproline inside mitochondria and the primary source of 4-hydroxyproline is collagen (dietary and endogenous).^18^ Hence, high levels of 4-hydroxyglutamate could result from increased collagen turnover and the release of 4-hydroxyproline. However, high levels could also result from decreased metabolism of 4-hydroxyglutamate. Its transamination by the mitochondrial enzyme Glutamic-Oxaloacetic Transaminase 2, GOT2, yields glutamate and 4-hydroxy-2-oxo-glutarate.^19^ Finally, it is well recognized that control of placental exchange of glutamate and glutamine has an important role in promoting normal pregnancy^20^ and it is possible that the association between 4-hydroxyglutamate and pre-eclampsia is due to a currently unrecognized metabolic pathway.

Although further studies will be required to delineate the mechanism linking maternal serum levels of 4-hydroxyglutamate to pre-eclampsia, the evidence supporting the association is strong. First, 4-hydroxyglutamate was identified on the basis of its association with term pre-eclampsia using the 12-, 20- and 28-wkGA samples. The association was much stronger in the initial validation stage at 36 wkGA than at earlier gestational ages. By using analysis of the cases of term pre-eclampsia to identify candidate remote markers of the disease, we reduced the number of metabolites being tested in our pre-term cases to just two, namely 4-hydroxyglutamate and C-glycosyltryptophan. The association between 4-hydroxyglutamate and pre-term pre-eclampsia was strong at all three gestational time points (i.e. 12, 20 and 28 wkGA). The results from these internal validation steps suggest the associations are unlikely to be chance findings. Finally, the generalizability of the finding was confirmed by external validation for the association with pre-eclampsia leading to either pre-term or term birth in the BiB cohort. The populations of the POP study and the BiB study differ profoundly in terms of parity, ethnicity and socio-economic status. The observation that the association was comparable in two such dissimilar populations provides strong evidence for the generalizability of the current findings. It is also highly plausible that true associations would be apparent in diverse human populations.

Although the focus of the current analysis was on first-trimester predictors of pre-eclampsia leading to pre-term delivery, prediction of pre-eclampsia in late pregnancy is also important. In women presenting with signs or symptoms, testing can inform the use of expensive and potentially harmful interventions, such as hospital admission, administration of steroids, transfer to a tertiary unit or delivery.^21^ In asymptomatic women, the estimated risk could be used to inform the intensity of antenatal surveillance.^11^ In late pregnancy, the ratio of two biomarkers, sFlt-1 and PlGF, has been shown to have high negative predictive value in the context of women presenting with symptoms or signs of pre-eclampsia.^21^ However, the sFlt-1:PlGF ratio is not yet recommended as a ‘rule-in’ test for the disease.^22^ Moreover, when combined with maternal risk factors as a screening test for the disease in later pregnancy in nulliparous women, about 25% of the population fall into an indeterminate category of risk.^11^ We found that 4-hydroxyglutamate enhanced prediction of pre-term pre-eclampsia when measured at 20 and 28 wkGA and of term pre-eclampsia when measured at 36 wkGA. Hence, measurement of this metabolite might have utility in the prediction of pre-eclampsia in later pregnancy, but improvement in prediction would have to be assessed in further studies. In the present study, we analysed pre-term and term pre-eclampsia cases separately to reduce the risk of false discovery. However, there are significant differences in the pathophysiology of pre-term and term disease^23^ and future studies could also address differences in metabolite levels directly comparing pre-term and term cases. Additional studies might also address whether 4-hydroxyglutamate is also associated with the risk of other cardiovascular or renal conditions outside pregnancy, given that women with pre-eclampsia have an increased risk of both in later life.^24^^,^^25^

It is well recognized that, in the application of ‘omics’ technologies, there is the potential for the generation of false-positive results. Typically, the number of potential predictors of disease is very large in relation to the number of cases studied, which leads to an increased risk of over-optimistic prediction of associations.^15^^,^^26^ In addition to the number of predictors, the out-of-sample performance of the prediction model depends on the total sample size and the events fraction, i.e. the proportion of cases in the sample.^27^ The present study was relatively large, although the ratio of cases of term pre-eclampsia to candidate metabolites was <1. We overcame this issue by using multiple sampling time points in the POP study and by performing external validation in the BiB study (Figure 1).

A recent review of 28 small studies on the metabolomics of pre-eclampsia could not identify a single validated metabolite demonstrating utility as a pre-eclampsia biomarker.^28^ Included in the review was a metabolomic study of 50 cases of pre-term pre-eclampsia and 108 controls that identified five possible biomarkers,^29^ although none of these was associated with pre-term pre-eclampsia in the POP study (data available from authors on request). These were most likely false discoveries due to a single time frame of blood sampling and a small total number of cases, i.e. 50 blood samples from 50 cases. In contrast, we analysed the results from 194 cases and exploited multiple temporally separated sampling points from the same women. The rigorous approach to selection likely explains the fact that we could replicate the association in a markedly different population.

In a review article published over 10 years ago, Carty and colleagues^30^ suspected that it was unlikely that a single marker would turn out to accurately predict pre-eclampsia. Instead, a combination of different types of risk factors would have to be considered. Addition of a single weakly predictive biomarker into an existing prediction model might not result in a clinically meaningful improvement in prediction, as has been shown, e.g. in the field of cardiovascular epidemiology.^31^ However, even a moderate increase in the AUC combined with carefully chosen thresholds might help in reclassifying women into risk categories and in targeting antenatal monitoring better.

## Conclusions

We conclude that 4-hydroxyglutamate is a novel biomarker for pre-eclampsia. The strength of association means that it may enhance prediction of the disease in the clinical contexts where protein biomarkers are already employed.

## Supplementary Material

dyz098_Supplementary_Information
