# Supplementary material for: 4-Hydroxyglutamate is a novel predictor of pre-eclampsia
Source: Int J Epidemiol. 2019 May 16;49(1):301–11. doi: 10.1093/ije/dyz098 (PMC7124498; doi:10.1093/ije/dyz098)
Supplement: dyz098_Supplementary_Information [file dyz098_supplementary_information.docx]

**Supplementary Information for Sovio U, McBride N, Wood AM, Masconi KL, Cook E, Gaccioli F, Charnock-Jones DS, Lawlor DA, Smith GCS. 4-hydroxyglutamate is a novel predictor of preeclampsia.**

**Table of Contents**

**1. Metabolomic profiling**

**2. Analysis plan for current manuscript**

**3. Case-cohort study outline (Appendix to the Analysis plan for current manuscript)**

**4. Supplementary Tables**

**5. STARD checklist**

**6. Supplementary References**

**1. Metabolomic profiling**

Metabolomic analysis of the serum samples was performed by Metabolon, Inc. (Durham, North Carolina, USA) using a modification of a previously described ultra-performance liquid chromatography (UPLC) mass spectrometry (MS) method.[^1^](#_ENREF_1)^,^[^2^](#_ENREF_2)

Samples were extracted by methanol and run by four separate UPLC-MS/MS methods:

(1) reverse phase (RP) with positive ion mode electrospray ionization (ESI) optimized for hydrophilic compounds. The extract was gradient eluted from a C18 column (Waters UPLC BEH C18-2.1x100 mm, 1.7 µm) using water and methanol, containing 0.05% perfluoropentanoic acid (PFPA) and 0.1% formic acid (FA);

(2) RP with positive ion mode ESI optimized for more hydrophobic compounds. The extract was gradient eluted from the same C18 column using methanol, acetonitrile, water, 0.05% PFPA and 0.01% FA;

(3) RP/UPLC-MS/MS method with negative ion mode ESI. The extract was gradient eluted from the column using methanol and water with 6.5mM Ammonium Bicarbonate at pH 8;

(4) hydrophilic interaction liquid chromatography (HILIC) column with negative ion mode ESI. The extract was gradient eluted using water and acetonitrile with 10 mM ammonium formate, pH 10.8.

The instrument configuration, data acquisition, and metabolite identification and quantitation were as previously described (see references cited above). Essentially, the structure of metabolites were identified by matching the ion features (retention time, molecular weight (m/z), MS fragmentation pattern, preferred adducts, and in-source fragments) in the experimental samples to a reference library of chemical standard entries. The confidence of this metabolite identification met most stringent tier 1 criteria defined by Schrimpe-Rutledge et al.[^3^](#_ENREF_3) Peaks were quantified using area-under-the-curve of primary MS ions. To adjust for instrument batch effects for each run day, the raw ion counts for each metabolite were divided by the median value for the run day. Missing values were assumed to be the result of falling below the detection sensitivity, and thus were imputed with the minimum detection value based on each metabolite.

The serum samples were frozen on the day blood was obtained and stored at minus 80C. They were thawed once, aliquoted, frozen again and stored again at minus 80C. They were sent to the facility on dry ice with continuous assessment of temperature until receipt. Analysis was performed in batches of 36 samples. A total of 3,200 samples were analysed, hence there were 89 batches (88 of them containing 36 samples and one containing 32 samples). The composition of samples in each batch was planned in advance. All samples from a given woman were run in the same batch. However, each batch was designed to minimize the potential for associations with disease through batch effects. Hence, on average, about two thirds of the samples in a batch belonged to women who had one or more adverse outcome and about one third of samples belonged to women who had no adverse outcomes.

**2. Analysis plan for current manuscript**

**Analysis plan for assessing the association between maternal serum metabolites and the risk of preeclampsia in the Pregnancy Outcome Prediction study (PMID 19019223 & 26360240).**

1. Aim

To determine the relationship between maternal serum metabolites at different gestational ages and the risk of preeclampsia using data from the Pregnancy Outcome Prediction (POP) study (PMID 19019223 & 26360240).

2. Study design

A case-cohort design within the POP study is used, as described in the Appendix to this analysis plan.

3. Metabolites

Out of the 3,200 samples sent to Metabolon (Appendix), 3,196 samples from 923 women processed successfully. 1,193 metabolites (untargeted) measured from each sample, 837 of them identified (known structural identity) and 356 unidentified (unknown structural identity). Output was received in the format of 1) raw metabolite data, 2) scaled imputed data. Scaled imputed data will be used in the present analysis, transformed to improve normality as required. Transformed metabolite values will be reported as z scores when treated as continuous variables. In addition, quintiles of metabolite values will be calculated and the extreme quintile (highest or lowest) or decile (highest or lowest) will be compared with quintiles 2 to 4 or deciles 2 to 9, respectively. The thresholds for quintiles and deciles will be calculated using the random sample of the whole cohort without exclusions (n=325, see Appendix to this analysis plan).

4. Preeclampsia

All definitions of outcome will be based on the 2013 ACOG Guideline (PMID 24150027), see Sovio et al, Hypertension 2017, PMID 28167687). Preeclampsia with preterm delivery and preeclampsia with term delivery (all non-superimposed + severe superimposed) will be analysed separately. Term preeclampsia cases will be compared with women from the sub-cohort who delivered at term without experiencing any preeclampsia. Preterm cases will be compared with the all the women in the sub-cohort who did not experience preterm preeclampsia. As a sensitivity analysis, we will exclude women who developed any term preeclampsia from the comparison group in the analysis of preterm preeclampsia.

5. Exclusion criteria

The following women will be excluded from all analyses: (i) no metabolite measurement at any GA, (ii) therapeutic termination of pregnancy, (iii) miscarriage or intrauterine death <23 wkGA, (iv) withdrew from study, (v) no outcome data. Additionally, non-severe superimposed term preeclampsia will be excluded from the sub-cohort in the analyses of term preeclampsia (this was the only preeclampsia phenotype not defined as one of the outcomes in the case-cohort design but some of these women were included in the sub-cohort).

6. Analytic approach

We will fit longitudinal linear mixed models to determine which metabolites differed in the maternal serum at 20 and/or 28 weeks of gestational age (wkGA) in the preeclampsia cases born at term and controls born at term. The analysis will be confined to the 837 metabolites of known identity. Transformed continuous metabolite values will be treated as outcomes in this analysis. Gestational age at the metabolite measurement is the independent variable in this model. We will test for an interaction between term preeclampsia and gestational age. We will calculate interaction P value for each metabolite from the composite Chi-squared test (two-sided) for the 20/28 week measurements. We will test for an excess of low P values using a one-sample Kolmogorov-Smirnov test against the theoretical distribution that the P values for the 837 values from identified metabolites are randomly distributed. If this test suggests an excess of low P values, we will select the 100 metabolites with the lowest P values for further study.

We will then validate the result using the metabolite level at 36wkGA from the same women, by fitting a regression model between the metabolite and term preeclampsia status. We will select the metabolites with a validation P<0.0005 for further analysis (i.e. apply Bonferroni correction for 100 comparisons, 0.05/100).

Validated metabolites will be included as continuous variables in a forward stepwise logistic regression model (p<0.05 for entry and p<0.1 for removal) to predict term preeclampsia. These models will include the sFlt-1:PlGF ratio at 36wkGA, maternal age and body mass index (BMI) at 12wkGA.

Next we will fit logistic regression models for each of the selected metabolites plus the sFlt-1:PlGF ratio at 36wkGA and compare the extent to which each metabolite on its own improves the prediction of the risk of preeclampsia at term. The area under the ROC curve (AUC) will be calculated using 1000-fold bootstrapping to simulate out of sample prediction. Having identified the single metabolite which causes the highest increase in AUC we will then add the second most informative metabolite to the model. We will continue to add metabolites where they increase the AUC by more than 0.01 compared to the model lacking the metabolite. The metabolites which achieve this will be defined as those which are independently predictive of term preeclampsia. These metabolites will then have a second process of validation using the cases of preterm preeclampsia.

Univariate analysis will be performed for each metabolite at 12, 20 and 28wkGA in relation to preterm preeclampsia. This will involve (i) a plot of the proportion of cases of preterm preeclampsia versus quintiles of the metabolite, (ii) calculation of the AUC for the metabolite as a continuous variable, (iii) a univariate logistic regression model of the metabolite as a continuous variable (appropriately transformed and expressed as a z score). Validation will be determined by the P value for the coefficient for the metabolite derived from the logistic regression model. Assessment of statistical significance will involve a Bonferroni adjustment of the P value threshold to account for the number of metabolites selected for validation. If >10 metabolites were eligible for validation, we will only study the top 10 based on the increase in AUC described above.

We will then assess the capacity of each of the validated metabolites to improve clinical prediction of preterm preeclampsia at the three time points when compared with estimation based on maternal characteristics. The association between maternal characteristics and the risk of preterm preeclampsia will employ the model described in Rolnik et al, NEJM 2017 (PMID 28657417). The output of their competing risks model is the predicted gestational age when preeclampsia would occur (referred to a PGAPE hereafter), hence, higher values reflect lower risk. We will employ the estimate based on the maternal characteristics alone (described in Table 1 of their Supplementary Appendix). The statistical significance of metabolites will be assessed using the likelihood ratio test comparing two models: (i) PGAPE plus the metabolites, and (ii) PGAPE on its own. The effect of the metabolites on clinical prediction of disease will be assessed (i) by the change in AUC caused by adding the metabolites (where all AUCs are calculated using 1000-fold bootstrapping to simulate out of sample prediction), and (ii) the sensitivity and positive predictive value for a 5% or 10% screen positive rate.

Finally, the association with the metabolites (statistical significance and effect on clinical prediction, assessed using the same methods described above) will be compared with maternal characteristics PLUS existing protein biomarkers. At 12 weeks, the following three models will be compared: (i) PAPP-A, PlGF and PGAPE, (ii) the metabolites and PGAPE (iii) PAPP-A, PlGF, the metabolites and PGAPE. At 20 and 28 weeks, the following five models will be compared: (i) sFLT1/PlGF ratio on its own, (ii) PGAPE on its own, (iii) sFLT1/PlGF ratio and PGAPE, and (iv) sFLT1/PlGF ratio, PGAPE and the metabolites, (v) sFLT1/PlGF ratio and the metabolites.

**3. Case cohort study design (Appendix to the Analysis plan for current manuscript)**

The aim of the study is to determine the association between maternal serum levels of metabolites and the risk of 10 categories of adverse pregnancy outcome. In each analysis, we will compare the cases with the given outcome of interest to a comparison group. The study uses a case cohort design. i.e. there is a randomly selected sub-group of the whole cohort (sub-cohort). Hence, there is overlap between the sub-group and the cases (the sub-group includes 46 participants who also experienced one of the 10 categories of adverse outcome). In the analysis of the given outcome, these women should be treated as cases. However, in the analyses of the other adverse outcomes which the women did not experience, they should be treated as controls.

**Table. Number of samples from cases of adverse outcome**

| **Outcome** | **N(samples)** | **N(women with ≥1 samples)** |
| --- | --- | --- |
| Pre-eclampsia with preterm delivery | 84 | 29 |
| Birth weight <10^th^ percentile with preterm delivery | 119 | 40 |
| Gestational diabetes requiring drug treatment* | 255 | 90 |
| Gestational diabetes requiring diet treatment* | 280 | 96 |
| Spontaneous preterm delivery | 342 | 113 |
| Severe, non-superimposed pre-eclampsia, term | 282 | 78 |
| Birth weight <10^th^ percentile with ultrasonic evidence of fetal growth restriction | 343 | 88 |
| Severe, superimposed pre-eclampsia, term delivery | 128 | 36 |
| Non-severe, non-superimposed pre-eclampsia, term | 179 | 51 |
| Birth weight <3^rd^ percentile, delivered at term | 462 | 120 |
| **Total number accounting for overlaps** | **2153** | **644** |

*Samples at 12/20/28 weeks only, gestational diabetes excluding pre-existing diabetes.

The cohort includes 4212 women who completed the study. After excluding miscarriages, fetal deaths prior to 23 weeks and terminations (total n=29) and women who did not have any blood samples for analysis (n=6), there were 4177 women in the cohort. % of total cohort defined as cases = 644/4177 = 15.4%. Total cases = 644 women with 2153 samples after accounting for overlaps (overlaps explain why the total at the bottom of the first column [2153] is less than the sum of the cells above [2474]). To select a control cohort where anyone could potentially develop any of the outcomes, we had to include all women with one or more serum sample available to the pool we draw the random sample from. Hence, some of the comparator group had already been selected as cases. Figure illustrates overlap.


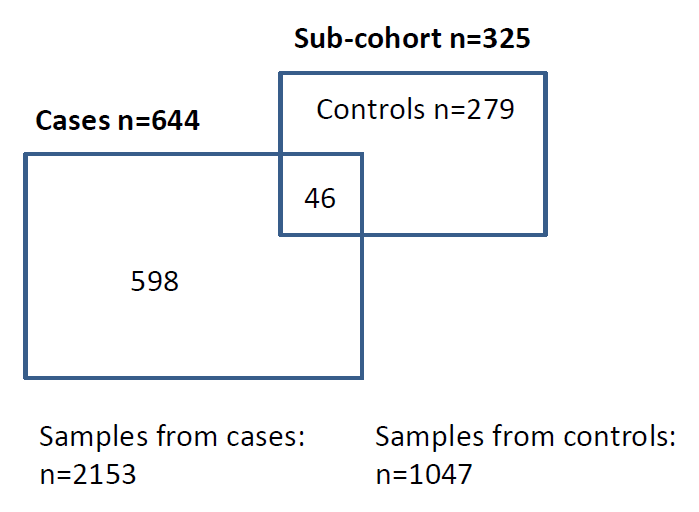


**Figure. Case cohort study design for the analysis of metabolites in the Pregnancy Outcome Prediction study.**

**4. Supplementary Tables**

**Supplementary Table 1. Known metabolites with different maternal serum levels by term preeclampsia, included into top 100 metabolites by *P* value at 20/28 weeks of gestational age (wkGA), validated using a 36wkGA measurement at P<5×10^−04^.**

| **Metabolite** | ***P* value**  **20-28 wkGA** | ***P* value**  **36wkGA** |
| --- | --- | --- |
| **C-glycosyltryptophan** | **0.0092** | **9×10^−16^** |
| N-acetylphenylalanine | 0.014 | 2×10^−11^ |
| 1-palmitoyl-2-palmitoleoyl-GPE (16:0/16:1)* | 0.0032 | 2×10^−10^ |
| Alpha-ketoglutarate | 6×10^−06^ | 2×10^−10^ |
| Glycerol | 0.0016 | 3×10^−10^ |
| Progesterone | 0.017 | 5×10^−10^ |
| **4-hydroxyglutamate** | **2×10^−04^** | **3×10^−09^** |
| 1-palmitoyl-2-oleoyl-GPE (16:0/18:1) | 0.0035 | 6×10^−09^ |
| 1-palmitoyl-2-palmitoleoyl-GPC (16:0/16:1)* | 0.018 | 9×10^−09^ |
| Palmitoyl-oleoyl-glycerol (16:0/18:1) [2]* | 8×10^−04^ | 1×10^−08^ |
| Beta-hydroxyisovalerate | 0.031 | 1×10^−08^ |
| **1-palmitoyl-2-linoleoyl-GPE (16:0/18:2)** | **4×10^−05^** | **5×10^−08^** |
| 1-palmitoyl-2-arachidonoyl-GPE (16:0/20:4)* | 0.014 | 7×10^−08^ |
| 1-stearoyl-2-linoleoyl-GPE (18:0/18:2)* | 9×10^−06^ | 8×10^−08^ |
| N-acetylcitrulline | 0.0085 | 1×10^−07^ |
| N-acetylneuraminate | 0.022 | 1×10^−07^ |
| 1-palmitoyl-2-oleoyl-GPC (16:0/18:1) | 0.021 | 3×10^−07^ |
| N2,N2-dimethylguanosine | 0.013 | 4×10^−07^ |
| 1-stearoyl-2-oleoyl-GPE (18:0/18:1) | 9×10^−05^ | 9×10^−07^ |
| 1-palmitoleoyl-GPE (16:1)* | 0.0029 | 1×10^−06^ |
| N-acetyltyrosine | 0.0096 | 1×10^−06^ |
| Choline | 0.024 | 2×10^−06^ |
| Oleoyl-oleoyl-glycerol (18:1/18:1) [2]* | 0.011 | 3×10^−06^ |
| 1-palmitoleoylglycerol (16:1)* | 0.023 | 3×10^−06^ |
| Glucuronate | 0.017 | 6×10^−06^ |
| N-stearoyl-sphingosine (d18:1/18:0)* | 0.0010 | 1×10^−05^ |
| 7-methylguanine | 0.0059 | 7×10^−05^ |
| 1-stearoyl-2-dihomo-linolenoyl-GPC (18:0/20:3n3 or 6)* | 0.0042 | 1×10^−04^ |
| 1-stearoyl-2-arachidonoyl-GPE (18:0/20:4) | 0.0018 | 1×10^−04^ |
| Palmitoyl-oleoyl-glycerol (16:0/18:1) [1]* | 0.0049 | 2×10^−04^ |
| 1-stearoyl-2-docosahexaenoyl-GPE (18:0/22:6)* | 0.015 | 2×10^−04^ |
| N-acetylkynurenine (2) | 0.015 | 3×10^−04^ |
| 3-hydroxyhexanoate | 0.011 | 5×10^−04^ |

Metabolites (n=33) are sorted by *P* value at 36wkGA. Differences at 20wkGA or 28wkGA (composite hypothesis) between women who developed term preeclampsia vs. women who delivered at term without preeclampsia were tested using a Chi-squared test (testparm postestimation command in Stata). Validation at 36wkGA was performed using a t test. All associations reported in the table were positive i.e. metabolite levels were higher in the women with term preeclampsia than in the controls. *Indicates compounds that have not been officially confirmed based on a standard. Metabolites selected for further assessment (see the main manuscript) marked **in bold.**

**Supplementary Table 2. Spearman correlations between the metabolites which validated at 36 weeks of gestational age in term preeclampsia cases and their controls.**

Metabolites included in the multivariable model after a forward stepwise selection in addition to BMI, age (linear and quadratic term) and sFlt-1:PlGF ratio are marked **in bold.**

**Supplementary Table 3. Prediction of preterm preeclampsia 20wkGA.**

| **Model** | **AUC** | **Change in AUC** | ***P* value^a^** |
| --- | --- | --- | --- |
|  |  |  |  |
| PGAPE + sFlt1:PlGF + 4-hydroxyglutamate | 0.911 | - | - |
| PGAPE + 4-hydroxyglutamate | 0.885 | -0.026 | <0.0001 |
| PGAPE + sFlt1:PlGF | 0.889 | -0.022 | 0.050 |
| PGAPE | 0.870 | -0.041 | <0.0001 |
| 4-hydroxyglutamate | 0.731 | -0.180 | <0.0001 |
| sFlt1:PlGF | 0.666 | -0.245 | <0.0001 |
|  |  |  |  |

^a^Compared with full model using likelihood ratio test of nested logistic regression models. Where the model included more than one predictor, the AUC was corrected for optimism using 1000-fold bootstrapping. Abbreviations: AUC, area under the ROC curve; wkGA, weeks of gestational age; PAPP-A, pregnancy associated plasma protein A; PGAPE, predicted gestational age of preeclampsia[^4^](#_ENREF_4) (calculated at 12wkGA and reported for women who also had the sample available at 20wkGA); PlGF, placenta growth factor; sFLT1, soluble fms-like tyrosine kinase 1.

**Supplementary Table 4. Prediction of preterm preeclampsia 28wkGA.**

| **Model** | **AUC** | **Change in AUC** | ***P* value^a^** |
| --- | --- | --- | --- |
|  |  |  |  |
| PGAPE + sFlt1:PlGF + 4-hydroxyglutamate + C-glycosyltryptophan | 0.926 |  | - |
| PGAPE + 4-hydroxyglutamate + C-glycosyltryptophan | 0.878 | -0.048 | <0.0001 |
| PGAPE + sFlt1:PlGF | 0.913 | -0.013 | 0.048 |
| PGAPE | 0.853 | -0.073 | <0.0001 |
| 4-hydroxyglutamate + C-glycosyltryptophan | 0.765 | -0.161 | <0.0001 |
| sFlt1:PlGF | 0.818 | -0.108 | <0.0001 |
|  |  |  |  |

^a^Compared with full model using likelihood ratio test of nested logistic regression models. Where the model included more than one predictor, the AUC was corrected for optimism using 1000-fold bootstrapping. Abbreviations: AUC, area under the ROC curve; wkGA, weeks of gestational age; PAPP-A, pregnancy associated plasma protein A; PGAPE, predicted gestational age of preeclampsia[^4^](#_ENREF_4) (calculated at 12wkGA and reported for women who also had the sample available at 28wkGA); PlGF, placenta growth factor; sFLT1, soluble fms-like tyrosine kinase 1.

**Supplementary Table 5. The effect of the metabolites on clinical prediction of preterm preeclampsia assessed by sensitivity and positive predictive value for a 10% screen positive rate at the three gestational ages.**

|  |  | **Sensitivity (95% CI)** | | **PPV (95% CI)** | |
| --- | --- | --- | --- | --- | --- |
|  |  |  |  |  |  |
|  |  | **PGAPE only** | **PGAPE + metabolites^a^** | **PGAPE only** | **PGAPE + metabolites^a^** |
|  | 12wkGA | 51.7 (33.1 to 69.9) | 55.2 (36.1 to 72.8) | 6.1 (3.1 to 11.8) | 6.8 (3.4 to 13.1) |
|  | 20wkGA | 56.0 (35.3 to 74.8) | 56.0 (35.3 to 74.8) | 5.4 (2.7 to 10.6) | 5.4 (2.7 to 10.6) |
|  | 28wkGA | 54.2 (33.2 to 73.7) | 54.2 (33.2 to 73.7) | 5.1 (2.5 to 10.1) | 5.1 (2.5 to 10.1) |
|  |  |  |  |  |  |

^a^Metabolites include 4-hydroxyglutamate at 12wkGA and 20wkGA, and both 4-hydroxyglutamate and C-glycosyltryptophan at 28wkGA. Abbreviations: CI, confidence interval; wkGA, weeks of gestational age; PGAPE, predicted gestational age when preeclampsia would occur;[^4^](#_ENREF_4) PPV, positive predictive value.

**Supplementary Table 6.** Characteristics of the Born in Bradford study cohort in the metabolomics analysis of preeclampsia

|  |  |  |  |
| --- | --- | --- | --- |
| **Characteristic** | | **Preeclampsia cases**  **(N=25)** | **Preeclampsia controls**  **(N=953)** |
|  |  |  |  |
| **Maternal characteristics** | |  |  |
|  | |  |  |
| Age, years | | 25 (17 to 38) | 27 (15 to 44) |
|  |  |  |  |
| Height, cm | | 164 (154 to 174) | 162 (144 to 186) |
|  | Missing | 1 (4%) | 13 (1%) |
|  |  |  |  |
| BMI, kg/m^2^ | | 28.30 (18.55 to 42.35) | 25.40 (15.35 to 52.21) |
|  | Missing | 1 (4%) | 35 (4%) |
|  |  |  |  |
| Smoking | | 5 (20%) | 168 (18%) |
|  |  |  |  |
| Alcohol | | 11 (44%) | 320 (34%) |
|  |  |  |  |
| Education higher than A- level | | 5 (20%) | 213 (22%) |
|  |  |  |  |
| Deprivation, score | | 49.56 (29.76 to 55.22) | 44.65 (25.91 to 55.65) |
| Deprivation, rank | | 2262  (1380 to 8300) | 3307  (1311 to 10188) |
| Deprivation rank quintile | |  |  |
|  | 1 (most deprived) | 11 (44%) | 357 (37%) |
|  | 2 | 6 (24%) | 241 (25%) |
|  | 3 | 4 (16%) | 181 (19%) |
|  | 4 | 0 (0%) | 124 (13%) |
|  | 5 (least deprived) | 3 (12%) | 43 (5%) |
|  | Missing | 1 (4%) | 7 (1%) |
|  | |  |  |
| Parity | |  |  |
|  | 1st pregnancy | 12 (48%) | 339 (36%) |
|  | Previous pregnancies | 13 (52%) | 585 (61%) |
|  | Missing | 0 (0%) | 29 (3%) |
| White ethnicity | | 14 (56%) | 477 (50%) |
|  |  |  |  |
| Married | | 17 (68%) | 628 (66%) |
|  |  |  |  |
| Gestational diabetes | | 1 (4%) | 88 (9%) |
|  |  |  |  |
| **Birth outcomes** | |  |  |
|  | |  |  |
| Birth weight, g | | 3300 (2480 to 3560) | 3280 (2980 to 3620) |
|  |  |  |  |
| Gestational age, weeks | | 38 (37 to 40) | 39 (38 to 40) |
|  | Missing | 6 (24%) | 181 (19%) |
| Female fetal sex | | 13 (52%) | 554 (58%) |
|  |  |  |  |
| Mode of delivery | |  |  |
|  | Spontaneous (vaginal) | 10 (40%) | 637 (67%) |
|  | Caesarean (elective or emergency) | 3 (12%) | 107 (11%) |
|  | Induction of labour (medical or surgical) | 12 (48%) | 208 (22%) |
|  | Missing | 0 (0%) | 1 (<1%) |

Data are expressed as median (IQR) or n (%) as appropriate. Data are complete unless NA is specified. Only two ethnic groups were included in this analysis: White British and Pakistani. There are distinctive population differences between these groups. Alcohol measures were classified as % of women who drank alcohol during pregnancy or 3 months before. Smoking measures were classified as % who had smoked at all during pregnancy. Married is inclusive of those who are remarried. Gestational diabetes status was derived from OGTT results. Maternal characteristics were taken from at recruitment (24-28 weeks) or from examination of the clinical case record, or linkage to the hospital’s electronic databases. The weight measurement used in the BMI calculation was the mother’s first antenatal clinic (booking) weight. Preeclampsia was defined based on the International Society for the Study of Hypertension in Pregnancy criteria.[^5^](#_ENREF_5) Socio-economic status was quantified using the Index of Multiple Deprivation (IMD) 2010.[^6^](#_ENREF_6) Deprivation score is the combined sum of the weighted, exponentially transformed domain rank of the domain score, and higher values indicate more deprivation. Conversely, the most deprived area has the lowest rank and the least deprived area has the highest rank. A national reference distribution from 2010 was used to analyse the rank in quintiles (1=most deprived, 5=least deprived). Abbreviations: BMI, body mass index.

**5. STARD checklist**

|  | **Section & Topic** | **No** | **Item** | **Reported on page #** |
| --- | --- | --- | --- | --- |
|  |  |  |  |  |
|  | **TITLE OR ABSTRACT** |  |  |  |
|  |  | **1** | Identification as a study of diagnostic accuracy using at least one measure of accuracy  (such as sensitivity, specificity, predictive values, or AUC) | 3-4 |
|  | **ABSTRACT** |  |  |  |
|  |  | **2** | Structured summary of study design, methods, results, and conclusions  (for specific guidance, see STARD for Abstracts) | 3-4 |
|  | **INTRODUCTION** |  |  |  |
|  |  | **3** | Scientific and clinical background, including the intended use and clinical role of the index test | 5 |
|  |  | **4** | Study objectives and hypotheses | 5 |
|  | **METHODS** |  |  |  |
|  | *Study design* | **5** | Whether data collection was planned before the index test and reference standard  were performed (prospective study) or after (retrospective study) | 6-7 |
|  | *Participants* | **6** | Eligibility criteria | 6-7 and Appendix |
|  |  | **7** | On what basis potentially eligible participants were identified  (such as symptoms, results from previous tests, inclusion in registry) | 6-7 |
|  |  | **8** | Where and when potentially eligible participants were identified (setting, location and dates) | 6-7 |
|  |  | **9** | Whether participants formed a consecutive, random or convenience series | 6-7 |
|  | *Test methods* | **10a** | Index test, in sufficient detail to allow replication | 7-8 |
|  |  | **10b** | Reference standard, in sufficient detail to allow replication | 6-7 and Appendix |
|  |  | **11** | Rationale for choosing the reference standard (if alternatives exist) | 6-7 and Appendix |
|  |  | **12a** | Definition of and rationale for test positivity cut-offs or result categories  of the index test, distinguishing pre-specified from exploratory | Appendix |
|  |  | **12b** | Definition of and rationale for test positivity cut-offs or result categories  of the reference standard, distinguishing pre-specified from exploratory | 6-7 and Appendix |
|  |  | **13a** | Whether clinical information and reference standard results were available  to the performers/readers of the index test | 7 |
|  |  | **13b** | Whether clinical information and index test results were available  to the assessors of the reference standard | 6 |
|  | *Analysis* | **14** | Methods for estimating or comparing measures of diagnostic accuracy | 8-10 |
|  |  | **15** | How indeterminate index test or reference standard results were handled | 7-8 and Appendix |
|  |  | **16** | How missing data on the index test and reference standard were handled | 7-8 and Appendix |
|  |  | **17** | Any analyses of variability in diagnostic accuracy, distinguishing pre-specified from exploratory | 8-10 and Appendix |
|  |  | **18** | Intended sample size and how it was determined | Appendix |
|  | **RESULTS** |  |  |  |
|  | *Participants* | **19** | Flow of participants, using a diagram | Figure 1 |
|  |  | **20** | Baseline demographic and clinical characteristics of participants | Table 1 and Appendix |
|  |  | **21a** | Distribution of severity of disease in those with the target condition | N/A |
|  |  | **21b** | Distribution of alternative diagnoses in those without the target condition | Table 1 |
|  |  | **22** | Time interval and any clinical interventions between index test and reference standard | Table 1 |
|  | *Test results* | **23** | Cross tabulation of the index test results (or their distribution)  by the results of the reference standard | Figure 3 |
|  |  | **24** | Estimates of diagnostic accuracy and their precision (such as 95% confidence intervals) | Tables 2 & 3, Figure 4 and Appendix |
|  |  | **25** | Any adverse events from performing the index test or the reference standard | N/A |
|  | **DISCUSSION** |  |  |  |
|  |  | **26** | Study limitations, including sources of potential bias, statistical uncertainty, and generalisability | 14-15 |
|  |  | **27** | Implications for practice, including the intended use and clinical role of the index test | 15-16 |
|  | **OTHER INFORMATION** |  |  |  |
|  |  | **28** | Registration number and name of registry | N/A |
|  |  | **29** | Where the full study protocol can be accessed | 6 & Appendix |
|  |  | **30** | Sources of funding and other support; role of funders | 17 |
|  |  |  |  |  |

**6. Supplementary References**

1. Evans AM, Bridgewater BR, Liu Q, et al. High Resolution Mass Spectrometry Improves Data Quantity and Quality as Compared to Unit Mass Resolution Mass Spectrometry in High-Throughput Profiling. *Metabolomics*. 2014;4(2):132.

2. DeHaven CD, Evans AM, Dai H, Lawton KA. Software techniques for enabling high-throughput analysis of metabolomic datasets. In: Roessner U, editor. *Metabolomics*: IntechOpen; 2012.

3. Schrimpe-Rutledge AC, Codreanu SG, Sherrod SD, McLean JA. Untargeted Metabolomics Strategies-Challenges and Emerging Directions. *J Am Soc Mass Spectrom*. 2016;27(12):1897-1905.

4. Rolnik DL, Wright D, Poon LC, et al. Aspirin versus Placebo in Pregnancies at High Risk for Preterm Preeclampsia. *N Engl J Med*. 2017;377(7):613-622.

5. Brown MA, Lindheimer MD, de Swiet M, Van Assche A, Moutquin JM. The classification and diagnosis of the hypertensive disorders of pregnancy: statement from the International Society for the Study of Hypertension in Pregnancy (ISSHP). *Hypertens Pregnancy*. 2001;20(1):IX-XIV.

6. McLennan D, Barnes H, Noble M, Davies J, Garratt E, Dibben C. The English Indices of Deprivation 2010. London: Department for Communities and Local Government; 2011.
